# Tumor invasiveness is regulated by the concerted function of APC, formins, and Arp2/3 complex

**DOI:** 10.1016/j.isci.2024.109687

**Published:** 2024-04-08

**Authors:** Lautaro Baro, Rabeah A. Almhassneh, Asifa Islam, M. Angeles Juanes

**Affiliations:** 1 Cytoskeletal Dynamics in Cell Migration and Cancer Invasion Laboratory, Centro de Investigación Príncipe Felipe, 46012 Valencia, Spain; 2 School of Health and Life Sciences, Teesside University, Middlesbrough TS1 3BX, UK; 3 National Horizons Centre, Teesside University, Darlington DL1 1HG, UK

**Keywords:** Biochemistry, Biological sciences, Cancer systems biology, Cell biology, Natural sciences, Systems biology

## Abstract

Tumor cell invasion is the initial step in metastasis, the leading cause of death from cancer. Invasion requires protrusive cellular structures that steer the migration of leader cells emanating from the tumor mass toward neighboring tissues. Actin is central to these processes and is therefore the prime target of drugs known as migrastatics. However, the broad effects of general actin inhibitors limit their therapeutic use. Here, we delineate the roles of specific actin nucleators in tuning actin-rich invasive protrusions and pinpoint potential pharmacological targets. We subject colorectal cancer spheroids embedded in collagen matrix—a preclinical model mirroring solid tumor invasiveness—to pharmacologic and/or genetic treatment of specific actin arrays to assess their roles in invasiveness. Our data reveal coordinated yet distinct involvement of actin networks nucleated by adenomatous polyposis coli, formins, and actin-related protein 2/3 complex in the biogenesis and maintenance of invasive protrusions. These findings may open avenues for better targeted therapies.

## Highlights

• APC-driven actin nucleation plays a role in the formation of invasive protrusions
• Different actin nucleators coordinate the biogenesis of invasive protrusion
• Formins and Arp2/3 complex do not participate in the maintenance of matured protrusions
• Dynamics of leader cells emanating from spheroids depends on actin nucleated by APC

## Graphical abstract

**Figure:**
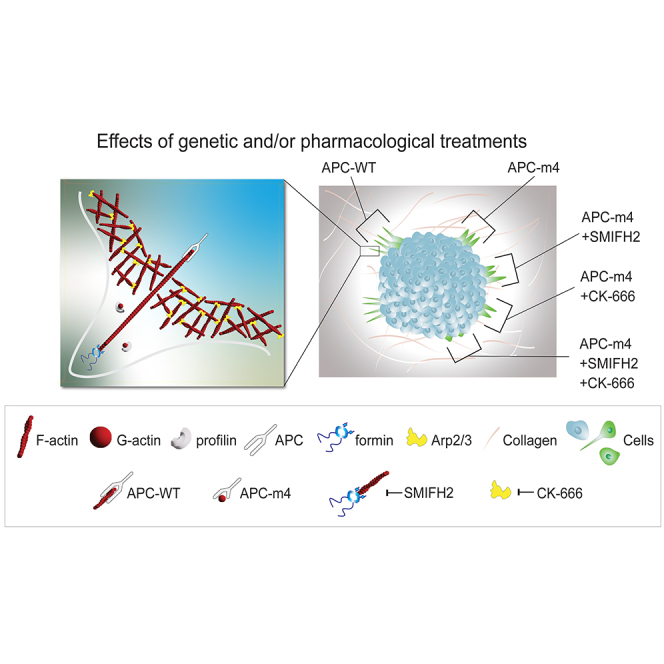

## Introduction

More than 90% of cancer deaths are associated with metastasis.^1^^,^^2^^,^^3^ The metastatic cascade is initiated by invasion which is mostly governed by reorganization of the actin cytoskeleton.^4^^,^^5^^,^^6^^,^^7^ On the one hand, the remodeling of the actin cytoskeleton results in the disruption of cell-cell adhesions that promotes loosening of malignant cells from the original tumor mass.^8^ On the other, actin remodeling can give rise to actin-rich protrusive structures called invadopodia that emanate from the tumor mass.^9^^,^^10^^,^^11^^,^^12^ Invadopodia are closely associated with networks of actin regulatory and adhesive molecules that together drive their elongation and maturation.^13^^,^^14^ Consequently, invadopodia undergo further transformation into larger actin-rich protrusions called pseudopodia that initiate cell body propulsion through the basement membrane and extracellular matrix (ECM). These will allow cells (individually or collectively) to navigate into the surrounding tissue and travel to other organs.^12^^,^^15^

Several actin nucleators participate in invadopodium-like or pseudopodium-like morphogenesis and activity.^7^^,^^16^ The actin-related protein (Arp)2/3 complex, together with other proteins including nucleation promoting factors (e.g., N-WASP/WAVE family members, cortactin), is responsible for dense 70° side-branched actin networks.^17^^,^^18^ Such networks known to promote lamellipodia are also essential for invadopodia and the development of pseudopods crucial for mesenchymal invasion.^17^ The increased expression of Arp2/3 complex subunits is evident in different cancers and correlates with poor prognosis, for example, the Arp2/3 subunit 5 (ARPC5) in head and neck cancer and lung squamous cell carcinoma, subunit 3 (ARPC3) and 4 (ARPC4) in pancreatic cancer, Arp2 and WAVE in breast cancer, and Arp2 and Arp3 in colorectal and gastric cancers.^17^^,^^19^^,^^20^

Formins belong to a family of actin nucleators that generate linear filaments.^21^^,^^22^ The diaphanous-related formins (DRFs) produce stress fibers and foster the development of actin-rich protrusions either at the leading edge to form filopodia or invadopodia-associated linear actin arrays.^23^^,^^24^ Particularly, DRF1, DRF2, and DRF3 are key during invadopodia formation for basement membrane degradation and invasion in breast cancer, as observed using 2D and thick 3D matrices.^24^ mDia1 controls the localization of the membrane type 1-matrix metalloproteinase in invadopodia and the consequent degrading activity and ECM remodeling.^25^ Formin-like2 (FMNL2), another member of DRFs, binds to cortactin to promote invadopodia formation and extracellular degradation by regulating actin polymerization and endosome motility.^26^ Expression levels of certain formins are associated with favorable or unfavorable prognosis depending on the type of cancer.^27^ For instance, elevated FMNL2-protein levels bound to cortactin may promote colorectal cancer (CRC) progression, whereas favorable prognosis has linked to high DIAPH1 levels in ovarian and renal cancers.

The tumor suppressor adenomatous polyposis coli (APC), a prototypical gatekeeper of CRC, nucleates linear actin filaments and this activity is critical for focal adhesion turnover, cell-cell adhesion dynamics, and collective cell migration.^28^^,^^29^^,^^30^^,^^31^^,^^32^^,^^33^ Moreover, a genome-wide screen using microarrays identified APC-associated RNAs accumulated at protruding pseudopodia in mouse fibroblasts.^34^ Single-molecule assays showed that APC strongly binds mRNAs and forms stable complexes that are transported along the cytoskeleton by kinesin motor proteins.^35^^,^^36^ Together, this raises the question whether APC-driven nucleation activity choreographs invadopodia- or pseudopod-like (herein finger-like protrusions) dynamics to promote cancer invasion, alone and/or in concert with other actin nucleation factors.

To begin to answer this question, we have dissected the involvement of different actin pools in cancer cell invasion using genetic and pharmacological tools combined with cell imaging techniques in the context of 3D intestine tumor spheroids, a preclinical model that shares functional similarity to solid tumors. Although intestinal spheroids have a round 3D structure, in contrast to 2D cell culture models, they lack architectural features such as shaped villi and crypts. Regardless, leader cells emanating from intestinal (round) spheroids can protrude forming 3D-finger-like structures that facilitate them to move away from the edge of the spheroid across a matrix. Crucially, we observed that the formation and elongation of 3D-finger-like structures was negatively affected upon “inactivation” of linear actin polymerized by APC. Those features were also affected upon inactivation of the Arp2/3 complex, mostly responsible to polymerize branched actin. However, inhibition of the linear actin pool polymerized by formins reduced the length of the 3D-finger-like structures but not number, compared to normal conditions. Interestingly, the inhibitory effect of each actin nucleator had a significant impact on protrusion number and length when paired to perturbation of other actin nucleator. Further, impairment of the three groups of actin nucleators was deleterious on the genesis of 3D-finger-like structures. These findings suggest that those actin nucleators act in independent pathways to drive invasiveness. Moreover, understanding the mechanistic contributions of specific cytoskeletal regulators in the facilitation of collective cell invasion will open new therapeutic avenues aimed at interfering with events preceding metastasis.

## Results

### Probing the contribution of actin nucleation mediated by APC in the formation of cancer cell invasive protrusions

**Figure 1:**
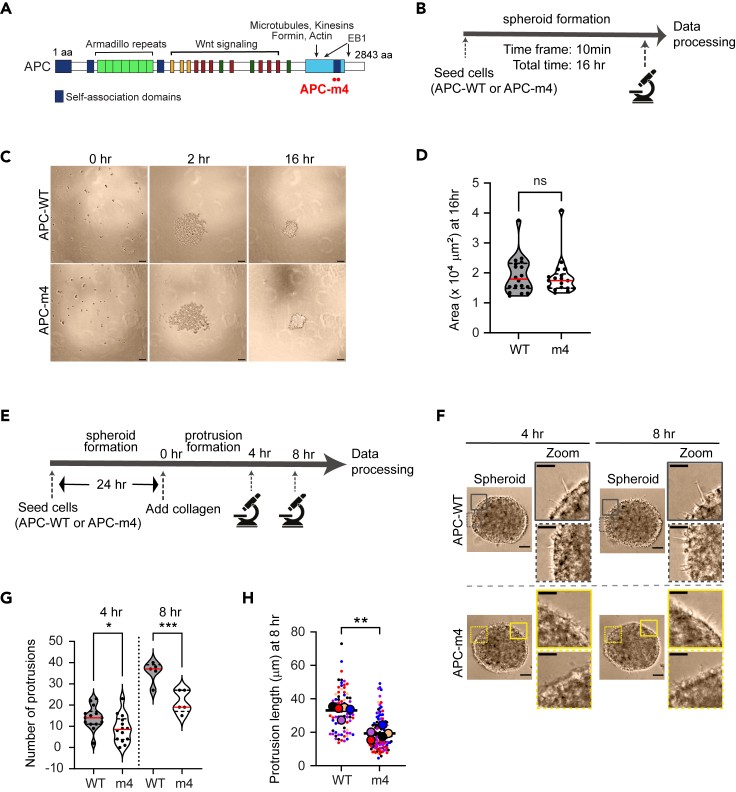
Expression of APC-m4 mutant does not affect spheroid remodeling All data are from LS174T cells stably expressing APC-WT or APC-m4. (A) Cartoon showing APC protein domains and location of the APC-m4 mutations. (B) Experimental regimen for monitoring spheroid formation. (C) Representative time-lapse images of cells treated as in (B). Scale bar, 100 μm. (D) Violin plot showing the spheroid area after 16 h of seeding cells, measured from images as those shown in (C). The solid red line is the median and the dotted lines are quartiles. Mann-Whitney U test was performed and “ns” denotes no significant difference. *N* = 3 replicates, *n* = 18 spheroids per condition. (E) Experimental regimen to induce invasive protrusions. (F) Representative images showing protrusions emanating from spheroids embedded in collagen for 4 h or 8 h. Insets correspond to two sections of the spheroid boundary to showcase protrusions. Scale bar, 100 μm, inset = 50 μm. (G) Violin plot showing the number of protrusions per spheroid at 4 h and 8 h after embedding in collagen. The solid red line is the median and the dotted lines are quartiles. The Mann-Whitney U test was performed to determine statistical significance. “∗∗∗” is *p* < 0.001 and “∗” is *p* < 0.05. One representative experiment is shown where *n* = 14 spheroids at 4 h, and *n* = 5 spheroids at 8 h per condition. (H) Violin plot showing the average length of invasive protrusions at 8 h. *N* = 5 independent replicates for each condition; n is individual protrusions used to quantify length: APC-WT = 83, APC-m4 = 131. Data are displayed as “Superplots” showing the mean of the different replicates (circles) and the distribution of “n length of protrusion analyzed” (color-coded dots) was superimposed as a beeswarm plot. Black solid line is the mean and paired two-tailed t-test was used to find the statistical differences. “∗∗” is *p* < 0.01.

To explore the impact of APC-driven actin nucleation on colorectal tumor cell invasion, we have engineered LS174T colorectal (adeno)carcinoma-derived epithelial cell lines stably expressing full-length wild-type APC (APC-WT) or a mutant version impaired in actin nucleation (APC-m4; Figure 1A^29^), on top of the endogenous copy of full-length APC. Regardless of the presence or absence of the endogenous APC, previous works showed that APC-m4 exerts a dominant-negative effect,^29^^,^^32^ similar to the cancer-linked C-terminal truncations of APC.^37^ Therefore, any ensuing phenotypes could be characterized in the presence of endogenous APC.^29^^,^^32^ APC-WT or APC-m4 individual clones were derived by limiting dilution and clones with similar expression levels of APC proteins were selected to continue this work (Figures S1A and S1B).

Stable LS174T APC-WT or APC-m4 cell lines were used to generate 3D cellular CRC spheroids, an *in vitro* cancer model retaining intrinsic microenvironment properties and functional similarities to solid tumors.^38^ To date, low-attachment surfaces have been widely used to induce cells in suspension to spontaneously undergo aggregation, thus mimicking tumors.^38^ After seeding, cells reach the center of the well by gravity, forming a circular cluster that ultimately undergoes reorganization into a compact spheroid. Accordingly, we seeded LS174T APC-WT or APC-m4 cells in 96-well ultra-low attachment culture plates to generate tumor spheroids. This process was visualized by time-lapse imaging microscopy throughout (Figures 1B and 1C). Briefly, both cell lines started to form clusters within the first hour after seeding (Figure 1C; Video S1). At 16 h from seeding, both LS174T APC-WT and APC-m4 cells made compact spheroids and exhibited similar areas, indicating that LS174T APC-WT or APC-m4 spheroids formed at the same pace (Figures 1C and 1D). Thus, APC-driven actin nucleation does not significantly contribute to cell aggregation and/or compaction of spheroids.

Video S1. Representative timelapse showing LS174T cells stably expressing APC-WT or APC-m4 forming 3D spheroids, related to Figure 1 Cells were imaged every 10 min for 16 h. Video playback is 5 frames per second. Scale bar = 100 μm.

Spheroids embedded in ECM-derived materials or matrices that mimic the ECM microenvironment can be used to study tumor cell invasiveness in three dimensions.^38^^,^^39^^,^^40^ For example, collagen provides a structural meshwork mimicking basement membrane and/or interstitial stroma ECM that invasive cells can breach and invade collectively after leader cells generate forces that protrude the plasma membrane into the surrounding environment while migrating away from the “compact tumor spheroid”. To address whether the actin pool nucleated by APC had any effect on the formation and/or elongation of invasive protrusions, spheroids were generated from LS174 APC-WT and APC-m4 as described previously. After 24 h from seeding cells—when spheroids were already formed—type I collagen was added on top of spheroids and their behavior was monitored using live imaging microscopy (Figure 1E; Video S2). After 4- and 8-h post-collagen addition, protrusions from both APC-WT and APC-m4 spheroids were observed (Figure 1F). Yet, quantitative analysis indicated that those were more numerous and longer in APC-WT than in APC-m4 spheroids (Figures 1G and 1H). At the 4-h time point, APC-WT generated on average 14 protrusions, while APC-m4 spheroids produced 9, an approximately 38% reduction in invasive protrusions in the mutant. Even though protrusions continued to develop over time in both cases, there was still a 40% decrease in invasive protrusions at the 8-h time point for the mutant relative to APC-WT spheroids (Figure 1G). Moreover, protrusion from APC-WT spheroids were approximately 41.70% longer than those in APC-m4 at the 8-h time point (average lengths of 33.10 and 19.30 μm, respectively; Figure 1H). To rule out that the decreased number of protrusions observed in LS174T APC-m4 relative to that in APC-WT spheroids could be due to a reduction in spheroid dimensions, we measured the area at the 8-h time point. However, no significant differences were found (Figure S1C).

Video S2. Representative timelapse showing LS174T spheroids stably expressing APC-WT or APC-m4 embedded in and invading in collagen, related to Figure 1 Cells were imaged live every hour for 4 h after 0 and 24 h after embedding in collagen, i.e., for a total of 28 h. Video playback is 1 frame per second. Scale bar = 100 μm.

Similar phenotype was also observed in an engineered HCT116 colorectal (adeno)carcinoma-derived epithelial cell line stably expressing full-length APC-WT or APC-m4 (Figures S1D and S1E^29^). Note that the HCT116 is a non-invasive cell line. However, in this cell line (as in others non-invasive cell lines), addition of the transforming growth factor β 1 (TGF-β1) into the cell medium has been a classic way to induce epithelial-to-mesenchymal transition (EMT).^41^ Together, these observations revealed a role of APC-driven actin nucleation in the efficient generation and elongation of invasive protrusions.

### The biogenesis of invasive protrusions relies on coordinated interplay between actin nucleation factors

**Figure 2:**
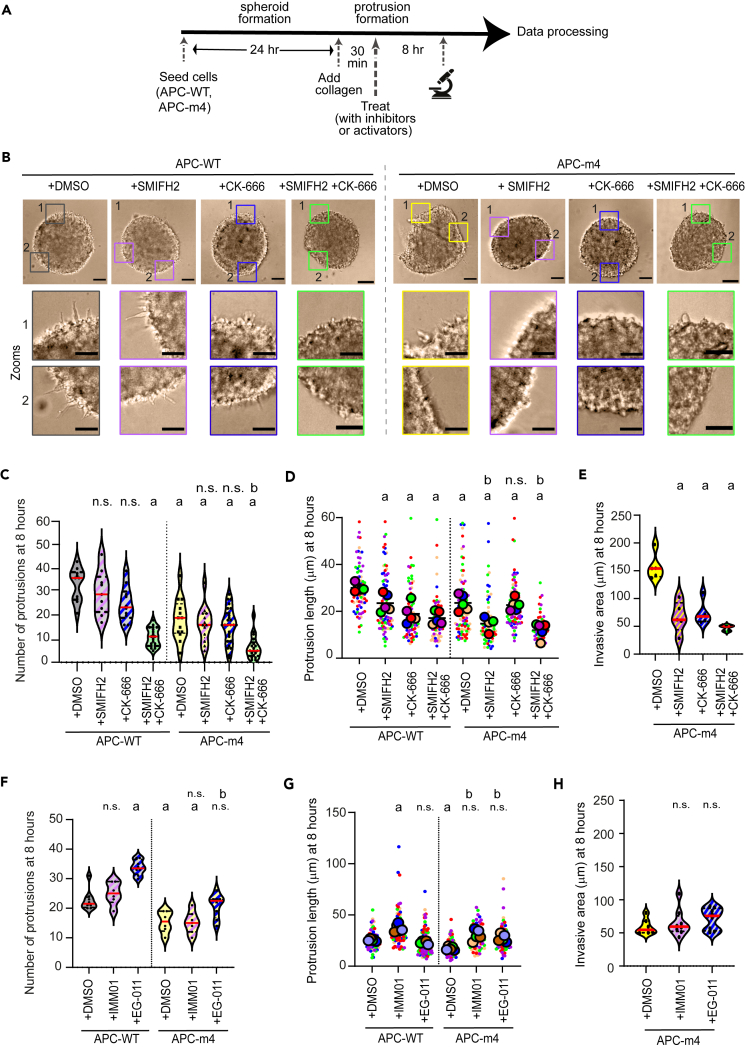
Effects of actin inhibitors in the formation of protrusions All data are from LS174T cells stably expressing APC-WT or APC-m4. (A) Experiment regimen for induction of invasive protrusions after pharmacological treatment. (B) Representative images showing spheroids embedded in collagen and treated with actin inhibitors. Insets correspond to selected sections of the spheroid periphery to view protrusions after 8 h of inhibitor treatment. Scale bar, 100 μm; inset = 50 μm. (C) Violin plot showing the number of protrusions per spheroid from images obtained as in (B). *N* = 3 replicates. The solid red line is the median and the dotted lines are quartiles. One-way ANOVA test was used to denote statistical significances. “a” denotes significant difference relative to APC-WT control (+DMSO) with *p* ranging from *p* < 0.001 to 0.05; and “n.s.” indicates not significant difference; and “b” is significant difference between APC-m4 (+DMSO) and other drug combination - relative to APC-m4, with *p* ranging from *p* < 0.001 to 0.05. (D) Violin plot showing the average length of invasive protrusions. *N* = 5 independent replicates for each condition; n is individual protrusions used to quantify length: APC-WT + DMSO = 78, APC-WT + SMIFH2 = 94, APC-WT + CK-666 = 86, APC-WT + SMIFH2 + CK-666 = 70, APC-m4 + DMSO = 99, APC-m4 + SMIFH2 = 67, APC-m4 + CK-666 = 88, and APC-m4 + SMIFH2 + CK-666 = 51 protrusions. Data are displayed as “Superplots” showing the mean of the different replicates (circles) and the distribution of “n length of protrusion analyzed” (color-coded dots) was superimposed as beeswarm plot. Black solid line is the mean and paired two-tailed t-test was used to find the statistical differences using *N* = 5 replicates. “a” is significant difference relative to APC-WT control (+DMSO) with *p* ranging from *p* < 0.001 to 0.05; and “b” is significant difference between combinations in APC-m4 but relative to APC-m4 control (+DMSO) with *p* ranging from *p* < 0.001 to 0.05. (E) Violin plot showing invasion area of spheroids after 8 h from embedding in collagen. *n* = 5 spheroids per condition. The solid line is the median and the dotted lines are quartiles. Ordinary one-way ANOVA test was performed for statistical significance. “a” is significant difference relative to APC-m4 control (+DMSO) with *p* ranging from *p* < 0.001 to 0.05. (F) Violin plot showing the number of protrusions per spheroid after 8 h from embedding in collagen. *N* = 8 replicates. The solid red line is the median and the dotted lines are quartiles. Statistical significance was derived from the Ordinary one-way ANOVA test. “a” denotes significant difference relative to APC-WT control (+DMSO) with *p* ranging from *p* < 0.001 to 0.05; and “n.s.” indicates not significant difference; and “b” is significant difference between APC-m4 (+DMSO) and other drug combinations - relative to APC-m4, with *p* ranging from *p* < 0.001 to 0.05. (G) Violin plot showing the average length of invasive protrusions. *N* = 5 independent replicates for each condition; n is individual protrusions used to quantify length: APC-WT + DMSO = 67, APC-WT + IMM01 = 74, APC-WT + EG-011 = 114, APC-m4 + DMSO = 78, APC-m4 + IMM01 = 74, and APC-m4 + EG-011 = 76 protrusions. Data are displayed as “Superplots” showing the mean of the different replicates (circles) and the distribution of “n length of protrusion analyzed” (color-coded dots) was superimposed as beeswarm plot. Black solid line is the mean and paired two-tailed t-test was used to find the statistical differences using *N* = 5 replicates. “a” is significant difference relative to APC-WT control (+DMSO) with *p* ranging from *p* < 0.001 to 0.05; and “b” is significant difference between combinations in APC-m4 but relative to APC-m4 control (+DMSO) with *p* ranging from *p* < 0.001 to 0.05. (H) Violin plot showing invasion area of spheroids after 8 h from embedding in collagen. *N* = 3 replicates; *n* = 8 spheroids per condition. The solid line is the median and the dotted lines are quartiles. Ordinary one-way ANOVA test was performed to find the statistical differences. “n.s.” indicates not significant difference.

We next sought to investigate the potential interplay between APC-driven actin nucleation, Arp2/3, and/or formins in the generation of finger-like structures. To this end, we first generated LS174T APC-WT spheroids, placed them in collagen, and shortly after subjected them to pharmacological treatment to perturb actin nucleation (Figure 2A). We used latrunculin-B — an actin polymerization inhibitor that sequesters free actin monomers, SMIFH2 — a pan-formin small-molecule inhibitor, or CK-666 — an Arp2/3 actin-branching inhibitor. After an 8-h treatment, spheroids were imaged and bright-field images were used to quantify protrusion number and length, as well as spheroid invasive and core area (Figures 2B–2E and S2; Table 1, and STAR Methods). Overall, latrunculin-B-treated APC-WT spheroids exhibited a marked reduction in protrusions, demonstrating these were actin-based extensions, in agreement with the literature (Figures S2A and S2B). Regarding the perturbation of nucleators, SMIFH2-treated APC-WT spheroids exhibited a modest reduction in the number of protrusions, and CK-666-treated APC-WT spheroids displayed a further reduction (Figure 2C). However, combined treatment (SMIFH2 and CK-666) caused a remarkable reduction in the number of protrusions in APC-WT spheroids. These observations pointed to a similar though synergistic role for formins and Arp2/3 in the generation of finger-like protrusions.

**Table 1:** Summary of cellular effects after genetic and/or pharmacological treatment

Results comparing with APC-WT + DMSO	Protrusion number	Protrusion length
APC-WT + SMIFH2	-	-
APC-WT + CK-666	- -	-
APC-WT + SMIFH2 + CK-666	- - -	- -
APC-WT + IMM01	+	+++
APC-WT + EG-011	+ + + +	-
APC-m4 + DMSO	- -	-
APC-m4 + SMIFH2	- -	- - -
APC-m4 + CK-666	- -	-
APC-m4 + SMIFH2 + CK-666	- - - -	- - - -
APC-m4 + IMM01	-	+
APC-m4 + EG-011	+ +	+

“-”: Modest reduction (though n.s.).

“- -“: Slight reduction (though n.s.).

“- - -”: Significant reduction.

“- - - -”: The most significant reduction.

“+”: Modest increase (though n.s.).

“+ +“: Slight reduction (though n.s.).

“+ + +”: Significant increase.

“+ + + +”: Remarkable increase.

In APC-m4 spheroids, the pan-formin inhibitor SMIFH2 caused a discrete reduction in the number of protrusions (Figure 2C). Similar effects were found upon treatment of APC-m4 spheroids with the Arp2/3 inhibitor CK-666 (Figure 2C). Remarkably, dual pharmacological treatment (SMIFH2 and CK-666 in conjunction) of APC-m4 spheroids almost abrogated protrusions. In addition, all spheroids subjected to pharmacological and/or genetic treatment displayed significantly shorter protrusions (Figure 2D). Moreover, the fact that SMIFH2 APC-m4 protrusions were even shorter than SMIFH2 APC-WT may indicate that at least certain formins may work in concert, yet in parallel, with APC to support the correct formation and/or elongation of protrusions (Figure 2D).

In line with this, APC-m4 spheroids treated with SMIFH2 or CK-666 inhibitors (alone and/or in combination) showed a marked reduction in the invasive area (measured as the difference between total and core area, see STAR Methods) when compared to untreated APC-m4 spheroids (Figure 2E). We confirmed that the differences found as a result of the respective treatments were not due to changes in spheroid core area (Figures S2D and S2E).

In contrast, APC-WT spheroids treated with IMM01 — a formin agonist, or EG-011 — a WASP activator, exhibited a higher number of protrusions than untreated APC-WT spheroids (Figure 2F). Among all these treatments, IMM01 treatment provoked significantly larger protrusions in APC-WT (Figure 2G). Moreover, IMM01 treatment rescued defective protrusion length, but not protrusion number or invasive area in APC-m4 spheroids. However, EG-011 treatment partially rescued both protrusion number and length but not invasive area in APC-m4 spheroids compared to untreated APC-m4 spheroids (Figures 2F and 2H). Therefore, the observed changes in the number and length of invasive APC-m4 protrusions may be due to active nucleation by formins and/or Arp2/3 complex. Taken together, and considering possible overlapping roles expected by the reported relationship between formins and APC in actin nucleation,^42^ our observations here demonstrate independent contributions among actin nucleators in the genesis of invasive finger-like protrusions.

### Maintenance of pre-existing protrusions do not require formins or Arp2/3 complex

**Figure 3:**
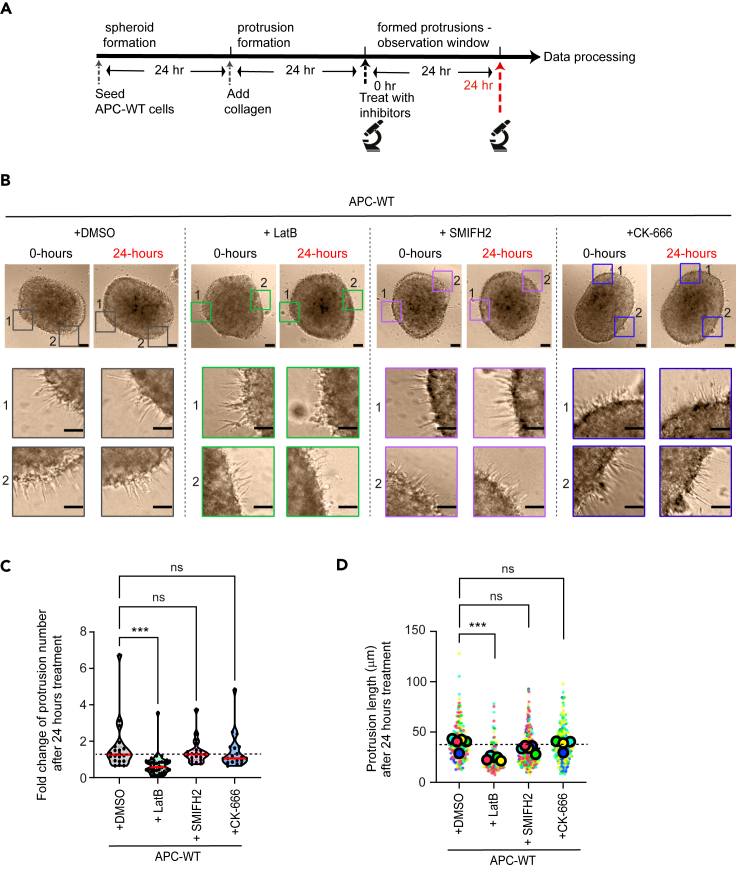
Effects of actin inhibitors in protrusion maintenance All data are from LS174T cells stably expressing APC-WT or APC-m4. (A) Experiment regimen for induction of protrusions, inhibitor treatment, and observation. (B) Representative images showing spheroids embedded in collagen and treated with inhibitors as indicated. Insets correspond to selected regions of the spheroid edge to show protrusions after 0 or 24 h of treatment. Scale bar, 100 μm; zoom = 50 μm. (C) Violin plot showing the fold change in the number of protrusions per spheroid for the different conditions after 24 h of inhibitor treatment. *N* = 5 replicates for each condition; n = total number of spheroids: APC-WT + DMSO = 20, APC-WT + Latrunculin-B = 36, APC-WT + SMIFH2 = 20, and APC-WT + CK-666 = 19. The solid red line is the median and the dotted lines are quartiles. Ordinary one-way ANOVA test was performed for statistical significance. “∗∗∗∗” is *p* < 0.0001, and “n.s” is not significant difference. (D) Violin plot showing the length of invasive protrusions. *N* = 5 independent replicates for each condition; n = individual protrusions used to quantify length: APC-WT+ DMSO = 167, APC-WT + Latrunculin-B = 147, APC-WT + SMIFH2 = 257, APC-WT + CK-666 = 202 protrusions. Data are displayed as “Superplots” showing the mean of the different replicates (circles) and the distribution of “n length of protrusion analyzed” (color-coded dots) was superimposed as beeswarm plot. Ordinary one-way ANOVA test was used to find the statistical differences using *N* = 5 replicates. “∗∗∗” is *p* < 0.001, and “n.s” is not significant difference.

In agreement with others, we found that Arp2/3 complex is critical for initiating protrusion formation while formins might have a more discrete role^24^^,^^43^ (Figure 2). We then asked whether those nucleators might be required to stabilize existing protrusions or even if their continued function might be required to sustain the formation of new protrusions before and after the basement membrane is breached. Accordingly, we allowed LS174T APC-WT spheroids to form protrusions for 24 h before treating with the inhibitors latrunculin-B, SMIFH2, or CK-666. Images were acquired before the addition of the drugs and 24 h post-treatment, and protrusions were quantified (Figure 3B). Latrunculin-B-treatment caused a reduction in the number of protrusions, as well as in their length, indicating that protrusions could be destabilized by inducing bulk actin depolymerization. In contrast, the treatment of established spheroids with either SMIFH2 or CK-666 did not change the number and/or length of protrusions observed prior to treatment (Figures 3C and 3D), suggesting that each actin pool on its own might be sufficient to retain protrusion numbers either by securing their stability (without new protrusions forming) or by adopting a set steady state (number of new protrusions offset by reduced lifespan).

### APC-driven actin nucleation guides protrusion dynamics of leader cells emanating from spheroids

**Figure 4:**
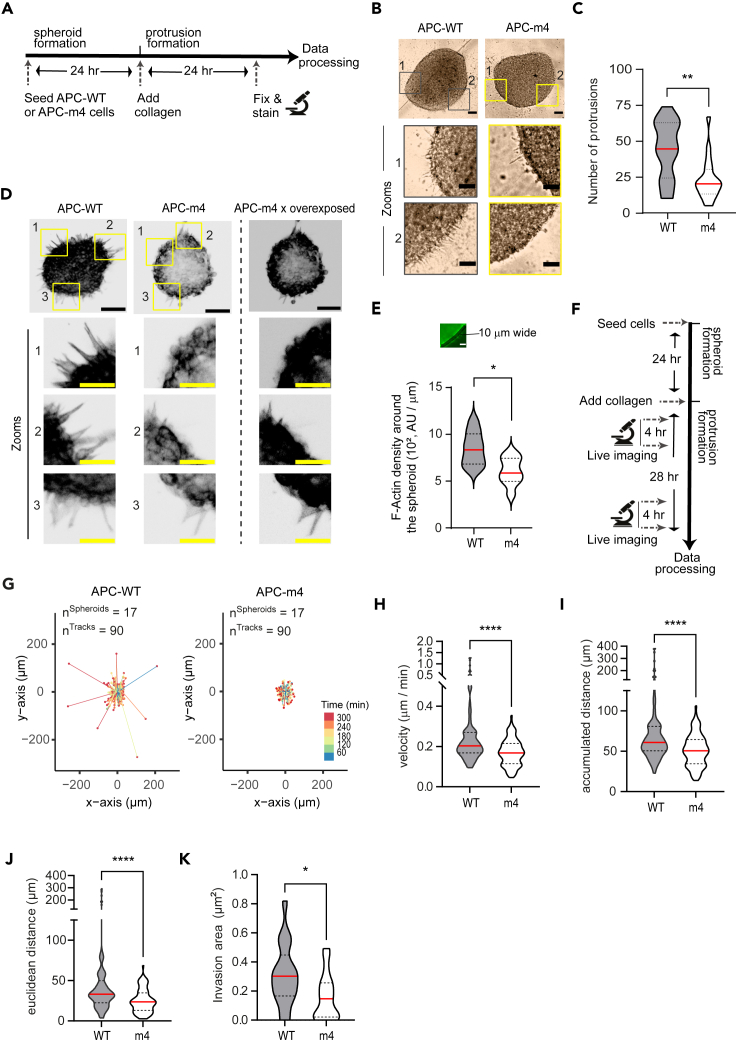
Expression of APC-m4 mutant disrupts invasion capabilities of colorectal cancer spheroids All data are from LS174T cells stably expressing APC-WT or APC-m4. (A) Experiment regimen. (B) Representative bright-field cell images after the complete regimen depicted in (A). Scale bar, 100 μm; inset = 50 μm. (C) Graph showing the number of protrusions emanating from spheroids over a 24-h observation window once embedded in collagen for 24 h. The red solid line is the mean, and the dotted lines are quantiles. *N* = 3 replicates; n = total number of spheroids: APC-WT = 17, and APC-m4 = 20. (D) Max projection of 5 planes showing F-actin fluorescence from spheroid images after the complete regimen depicted in (A). The two last columns show the same APC-m4 spheroid, being the first APC-m4 spheroid showed at the same scale of greys than APC-WT spheroid (on the left) but overexposed in the last column. Scale bar, 100 μm; inset = 50 μm. (E) Density of F-actin around 10 μm wide around the periphery of the spheroid. The red solid line is the mean and dotted lines are quartiles. *N* = 5 replicates. (F) Experiment regimen for monitoring protrusion formation and cell migration. (G) Representative traces of the migration paths of individual cells forming protrusions, moving out of the spheroids, are displayed in horizontal arrays. (H–J) Violin plots showing the velocity (H), accumulated distance (I), and Euclidean distance (J) of individual cells from the edge of the spheroid during a 4 h observation window after spheroid embedding in collagen. G–J: *N* = 3 replicates; *n* = 90 individual cells traced per condition. (K) Violin plot showing invasion area of spheroids after 28 h after embedding in collagen. *N* = 3 replicates; *n* = 17 spheroids per condition. The solid line is the median and the dotted lines are quartiles. Student’s t-test with Welch correction or Mann-Whitney U test was performed to find the statistical differences. “∗∗∗∗” is *p* < 0.0001, “∗∗” is *p* < 0.01, and “∗” is *p* < 0.05.

To investigate whether actin filaments nucleated by APC could play a role in the dynamicity of cancer cell protrusions, LS174T APC-WT or APC-m4 spheroids were formed for 24 h, embedded in collagen matrix, and allowed to form protrusions for a further 24-h. Protrusive spheroids were then fixed and stained with phalloidin to visualize F-actin. After fixation and staining, images were acquired and analyzed (Figure 4A). APC-WT spheroids exhibited multiple invading cell protrusions under bright-field microscopy. In agreement with Figure 1F, APC-m4 spheroids displayed a reduction of approximately 50% in the number of protrusions (Figures 4B and 4C). Under fluorescence microscopy, we observed a reduction in F-actin staining around the periphery of spheroids in APC-m4 compared to APC-WT spheroids (Figures 4D, 4E, and S2A).

We have previously demonstrated that expression of the APC-m4 mutant abrogates the directed movement of individual cells in chemotaxis assays, as well as the movement of a sheet of cells in wound assays.^29^^,^^32^ In addition, actin-associated networks have been implicated in steering actin-based structures during lamellipodium growth.^44^ Finally, invasion might be biased by a gradient field induced by the spheroid such that cells migrate radially.^45^ To test cell migration in our spheroid settings, we performed similar experiments but used live imaging to then track individual trajectories of cells emanating from protrusive spheroids generated from APC-WT versus APC-m4 cells after 24-h embedded in the collagen matrix (Figure 4F). We found that APC-m4 cells moved significantly slower (∼0.17 vs. ∼0.2 μm/s) and covered shorter distances (accumulated distance: ∼50.5 vs. ∼60.8 μm) relative to APC-WT cells (Figures 4G–4I). Moreover, the length of protrusions inferred from the Euclidean distance (i.e., the shortest distance between two points) was significantly shorter in APC-m4 versus APC-WT spheroids (∼23.6 vs. ∼33.1 μm; Figure 4J). Supporting this observation, APC-m4 spheroids exhibited an important reduction in the invasive area compared to APC-WT spheroids (Figure 4K, see also STAR Methods). These data suggest that actin nucleation mediated by APC is required for active invasive protrusion dynamics, which in turn could help guide leader cell protrusions to navigate the surrounding 3D matrix. Taken together, the data suggest that APC-dependent actin nucleation is required for robust cancer cell invasiveness.

## Discussion

Classic cancer therapies are based upon cytotoxic agents that mainly hit cell proliferation and/or promote programmed cell death (apoptosis).^46^^,^^47^^,^^48^ However, solid cancers are accompanied by local invasion of cells.^49^^,^^50^ The invasive and metastatic potential of tumor cells is responsible for most cancer deaths, and relapse is usually associated with more aggressive invasive phenotypes. Thus, it is of great importance to identify more effective and/or selective ways to stop cancer from spreading beyond its point of origin.^51^ Local invasion requires chemotactic migration of cancer cells, steered by the protrusive activity of the cell membrane and its attachment to the ECM. These processes are governed by the actin cytoskeleton.^52^^,^^53^ Inhibitors of actin polymerization/depolymerization have been proposed as “migrastatics” (anti-invasion and anti-metastatic).^54^ However, their use has been limited, given the potential impact on multiple aspects of actin function beyond migration, e.g., cell division, transport of organelles or vesicles, and metabolic rearrangements, among others.^54^^,^^55^

With the goal of developing strategies to block specific actin networks, this study explored the contribution of various actin nucleators in early stages of tumor cell invasiveness. Our findings highlighted the concerted requirement of APC, formins, and Arp2/3 complex to initiate finger-like protrusions and/or sustain their maturation. We demonstrated that the combined loss of branched and linear actin supported by Arp2/3 complex in combination with either APC or formins significantly prevents the formation of invasive protrusions. Protrusions were massively abolished upon loss of those three groups of actin nucleators. The fact that cells lacking either branched or linear actin can cope to form invasive protrusions, albeit defective in number and length, might indicate that their coordinated presence is indeed a requisite for optimal invasiveness. Likewise, concerted action by different actin populations has been described in 2D configurations, for example, for effective cell wound repair.^56^ In addition, the geometric organization contributed by different actin networks has been shown to generate force to push against the nucleation site to form and/or reinforce protrusive structures to continuously push while protrusions elongate and/or steer.^44^

Degradation of the ECM, achieved by invasive protrusions mechanically breaching the basement membrane, and cell migration are two important steps to promote tumor cell invasion and metastasis.^53^^,^^57^ Our findings here implicate APC-driven actin nucleation activity in these processes. By contrast, APC depletion in breast cancer cells appeared to increase their degradative potential in a 2D setting.^58^ This discrepancy might stem from the different experimental scenarios.^58^ Furthermore, depletion of a gene product might not be equated to the expression of a full-length separation-of-function mutant. With a multifunctional protein such as APC, the former will cause loss of multiple activities and interactions while the latter probes APC’s actin nucleation function without affecting other activities.^29^

We also found that impairment of APC-driven actin nucleation significantly affects the biogenesis of finger-like protrusions, compared with other actin nucleators tested here. Perhaps ECM or adhesive molecules are lower in APC-m4 spheroids, and the resulting protrusions are less robust. Although we have not investigated those possibilities in detail in spheroids, we have previously reported that individual cells expressing APC-m4 presented lower levels of focal adhesion proteins (including Paxillin, Zyxin, FAK, Src, and PAK kinases) and adherens proteins.^30^^,^^32^ Moreover, APC-m4 individual cells exhibited reduced levels of key signaling proteins from the tumor necrosis factor alpha and the TGF-β pathways that promote EMT (Figure S3B).^41^^,^^59^^,^^60^ These evidence together with the fact that (1) ECM regulates tumor cell invasion through integrins and FAK^57^^,^^61^ and (2) protrusion maturation requires activation and recruitment of proteases degrading the ECM^62^ make our hypothesis plausible. Alternatively, defective generation of actin arrays nucleated by APC might render actin networks less stiff and/or tense, thus compromising the integrity of existing protrusions. In addition, we found that lack of this actin population perturbed the steering of leader cells migrating away from intestinal tumor spheroids. Together, these findings uncover important roles for APC at early stages of tumor cell protrusions and invasiveness. Hence, targeting this pool of actin may slow cancer spread (metastasis) and increase survival in CRC patients.

These data might challenge the classical conception of APC as a tumor suppressor; yet considering the high prevalence of mutations spanning APC in cancer patients, *APC* status might provide a stratification factor in the prognosis of the disease. Moreover, this innovative strategy based on separation-of-function mutants could be expanded to produce a complete mutant toolkit to stratify aggressive and non-aggressive tumors to boost cancer surveillance and therapy selection.

### Limitations of the study

In this study, we have used intestinal spheroids as a model system, which have been widely used in cancer cell studies. However, spheroids are cultured in a “static environment” that does not fully represent the physiological microenvironment of the tissues. In addition, intestinal spheroids as the name says have spherical shape, losing the complex structure of the intestinal epithelium which is compartmentalized into crypts and villus structures to maintain homeostasis. Further, we have used genetic and pharmacological tools to assess response of actin nucleators, and it may be possible that such tools provoke subtle reactions or adaptation that could influence outcomes.

## STAR★Methods

### Key resources table

**Table:** 

REAGENT or RESOURCE	SOURCE	IDENTIFIER
**Antibodies**		

Rabbit anti-APC	Abcam	Cat# ab154906; RRID:AB_2861396
Goat anti-rabbit AlexaFluor-555	Thermo Fisher Scientific	Cat# A-21428, RRID:AB_2535849
NF-Kbeta p50 (H-119)x (Rabbit Polyclonal IgG)	Santa Cruz	Cat# Sc-7178x, RRID:AB_650211
Smad2/3 complex 8 (Mouse Monoclonal IgG2a)	Santa Cruz	Cat# Sc- 133098
Snail (Mouse Monoclonal IgG1)	Santa Cruz	Cat# Sc-271977, RRID:AB_2193048
Anti-mouse IgG (peroxidase antibody)	Sigma-Aldrich	Cat# A9044, RRID:AB_258431
Anti-rabbit IgG (peroxidase antibody)	Sigma-Aldrich	Cat# A0545, RRID:AB_257896

**Chemicals, peptides and recombinant proteins**		

FBS – Fetal bovine serum	Sigma-Aldrich	Cat# F9423
Antibiotics (Penicillin and Streptomycin)	Thermo Fisher	Cat#15140-122
DMEM- Dulbecco’s Modified Eagle Medium high glucose with pyruvate and L-Glutamine	Thermo Fisher Scientific	Cat# 41966029
Phosphate-Buffered Saline (PBS)	Sigma-Aldrich	Cat# P4417
Lipofectamine 3000	Thermo Fisher Scientific	Cat# L3000-015
G418	Thermo Fisher Scientific	Cat#10131035
Opti-MEM reduced serum medium	Thermo Fisher Scientific	Cat# 11058021
Levo L15 (Leibovitz)	Thermo Fisher Scientific	Cat# 21083027
Triton X100	Sigma-Aldrich	Cat# T8787-100ML
Tween20	Sigma-Aldrich	Cat# P1379-100ML
BSA	Sigma-Aldrich	Cat# A3059-10G
Complete protease-EDTA free inhibitor	Roche	Cat# 04693132001
RIPA buffer	Thermo Fisher Scientific	Cat# 89900
ECL Prime Western Blotting Detection Reagent	Cytiva	Cat# GERPN2232
Paraformaldehyde 16% - methanol free (PFA)	Thermo Scientific	Cat# 28908
Dimethyl sulfoxide (DMSO)	Thermo Scientific	Cat# D12345
AlexaFluor-488-Phalloidin	Invitrogen-Thermo Fisher Scientific	Cat# A12379
Collagen Type I, Rat Tail	Thistle Scientific	Cat#IB-50206
1N NaOH	Sigma-Merck	Cat# S2770-100ML
Sodium bicarbonate solution (NaHCO3)	Sigma-Merck	Cat# S8761-100ML
Latrunculin B	Abcam	Cat# ab144291
Formin FH2 domain inhibitor (SMIFH2)	Calbiochem-Merck	Cat# 344092-10MG
CK-666	Sigma-Merck	Cat# SML006-5MG
Diaphanous (mDia)-related Formin Agonist, IMM01	Sigma-Merck	Cat# 509583
Wiskott-Aldrich syndrome protein (WASP) activator, EG-011	MedChemExpress	Cat# HY-148683

**Experimental models: Cell lines**		

LS174T human colorectal cell line	ATCC-CL-188	RRID:CVCL_1384
LS174T APC-WT human colorectal cell line	This work	Juanes Lab
LS174T APC-m4 human colorectal cell line	This work	Juanes Lab
HCT116 APC-WT human colorectal cell line	(32)	Juanes Lab
HCT116 APC-m4 human colorectal cell line	(32)	Juanes Lab

**Recombinant DNA**		

Plasmid: APC-m4	(29)	Bruce Goode-US
Plasmid APC-WT	Addgene	Cat#16507

**Software and algorithms**		

LAS X Life Science Microscope Software version 3.5.5.19976	Leica	RRID:SCR_013673
Chemotaxis and Migration Tool version 2.0 software	Ibidi, Germany	RRID: SCR_022708
GraphPad Prism version 9	GraphPad Software	RRID: SCR_002798
Adobe Illustrator CC version 26.5	Adobe Illustrator (Adobe Systems)	RRID:SCR_010279
RStudio version 4.2 (ggplot2)	RStudio	RRID:SCR_000432
Python version 2.12	Python	RRID:SCR_008394
R script for tracking using ggplot2 in RStudio	This work	https://doi.org/10.5281/zenodo.8387899
Microsoft Excel version 16	Office365	RRID:SCR_016137
Fiji / ImageJ version 2.3	NIH – public domain	RRID:SCR_002285
Image Quant 10.2 analysis software	Cytiva	RRID:SCR_014246

**Other**		

AquaMount mounting medium	Thermo Fisher Scientific	Cat# 14-390-5
Circular Round cover glasses 0.18 mm	VWR International Ltd UK	Cat# 631-0153
Glass Slides	Academy	Cat# N/A141
Nunclon Sphera 96-well U-Bottom plates	Thermo Fisher	Cat# 174925
PVDF membranes 0.45 μm	Thermo Scientific	Cat# 88518
Dried semi-skimmed milk	Marvel	NA

### Resource availability

#### Lead contact

Requests for further information and reagents should be directed to and will be fulfilled by the Lead Contact, M.Angeles Juanes (majuanes@cipf.es).

#### Materials availability

This study has generated new cell lines which would be made available upon request to the lead contact M.Angeles Juanes.

#### Data and code availability

• All data reported in this paper will be shared by the lead contact upon request.
• The custom R script used in celltrackR and ggplot2 in RStudio to represent the single cell tracks has been deposited at Zenodo and is publicly available (version 1) as of the date of publication. https://doi.org/10.5281/zenodo.8387898.
• Any additional information required to reanalyze the data reported in this paper is available from the lead contact upon request.

### Experimental model and study participant details

LS174T (#ATCC-CL-188 from LGC/ATCC, RRID:CVCL_1384) expressing APC-WT or APC-m4 (generated in this study) and HCT116 (#91091005 from Merck, RRID:CVCL_0291;^29^) human colorectal cancer cell lines were grown in Dulbecco’s Modified Eagle’s Medium (DMEM high glucose with pyruvate and L-Glutamine (#41966029; ThermoFisher) supplemented with 10% Fetal Bovine Serum (FBS; # F9423; Sigma-Aldrich) and antibiotics (Penicillin and Streptomycin (#15140-122; ThermoFisher) at 37°C, 95% humidity, and 5% CO_2_.

Plasmid transfection was performed using Lipofectamine 3000 (#L3000-015; ThermoFisher) according to the manufacturer’s instructions. In addition to the standard protocol, after 4-6 hours, the transfection medium was removed and fresh pre-warmed DMEM was added to each well. For plasmid transfection in spheroids, the transfection media was changed after 14-16 hours.

LS174T colorectal cancer cell lines stably expressing APC-WT and APC-m4 were generated by transfecting plasmids encoding APC-WT (#16507; Addgene) or APC-m4.^29^ A plasmid encoding GFP-alone (#54759, Addgene) was used as transfection control. After plasmid transfection, cells were grown in medium containing 600 mg/ml G418 (#10131035; ThermoFisher) until single colonies were observed. Four colonies per condition were picked, and APC expression was confirmed using immunofluorescence assays. Cells were maintained in medium containing 200 mg/ml G418). Clones with comparable APC levels were used in subsequent experiments and for a maximum of 10 passages.

### Method details

#### Generation, embedding, and treatment of spheroids

To generate spheroids, a total of 500 LS174T or HCT116 colorectal cancer cells was resuspended in 200 μl of DMEM, seeded in an individual well of Nunclon Sphera 96-well U-Bottom plate (Thermofisher; Cat# 174925) and incubated at 37°C, 5% CO_2_, and 95% humidity for up to 24 hours.

To embed spheroids, collagen Type I (1.5 mg/ml rat tail tendon, Thistle Scientific; Cat#IB-50206) was thawed on ice; in the meantime, 150 μl of the total media were removed from each well containing pre-formed spheroids and 150 μl of a Matrix mix was added onto each pre-formed spheroid. The Matrigel mix consisted of 100.5 μl of 1X DMEM, 2.5 μl of 1M NaOH (Sigma-Merck; Cat# S2770-100ML), 2 μl of 7.3% NaHCO_3_ (Sigma-Merck; Cat# S8761-100ML), and 45 μl of Collagen Type I. Embedded spheroids were kept at 37°C, 5% CO_2_, and 95% humidity. One hour after embedding in collagen HCT116 spheroids, 10 ng/mL of the transforming growth factor-β (TFG-beta1; ThermoFisher, Cat# PHG9214) was added into the cell medium (for each well) to induce epithelial to mesenchymal transition (EMT) across the collagen matrix (i.e., to induce formation of protrusions and cell invasion).

To assess effects of actin inhibitors or activators in the formation of protrusive invasions, spheroids embedded in collagen for 30 minutes were then treated with one of the following either actin inhibitors: 1 μM Latrunculin-B (Abcam; Cat# ab144291), 20 μM SMIFH2 (Calbiochem-Merck; Cat# 344092-10MG), or 50 μM CK-666 (Sigma-Merck; Cat# SML006-5MG); or actin activators: 100 μM Diaphanous (mDia)-related Formin Agonist, IMM01 (Sigma-Merck; Cat# 509583) or 5 μM Wiskott-Aldrich syndrome protein (WASP) activator, EG-011(MedChemExpress; Cat# HY-148683). DMSO was used as a control. Note that both actin inhibitors and activators were diluted 1X DMEM at 1/1000 from stocks and used at their optimal working concentrations to avoid undesired anti-proliferative effects.^63^^,^^64^^,^^65^ Treated spheroids were incubated at 37°C, 5% CO_2_, and 95% humidity and then imaged at different time points.

To assess maturation of invasive protrusions upon pharmacological treatment, similar protocol was used, except that the spheroids were embedded in the collagen matrix for 24 hours - to allow the formation and/or extension of protrusions - and then treated with actin inhibitors for another 24 hours. Those spheroids were imaged right after treating with inhibitors and 24 hours post-treatment.

#### Western Blot

To determine protein levels, cells were pelleted and resuspended in RIPA lysis buffer (150 mM NaCl, 1% NP-40, 1% sodium deoxycholate, 0.1% SDS, 25 mM Tris, pH 7.6, and 1× Roche complete protease-EDTA free inhibitor mixture),.and incubated at 4°C for 30 min with vortexing every 2 min. Lysates were precleared by centrifugation at 15,300 x *g* for 20 min at 4°C, and the soluble protein concentration was determined by Bradford assay (Biorad, Hercules, CA). Equal amounts of lysate were resolved by SDS/PAGE (8-12% polyacrylamide) and transferred to PVDF membranes (Thermo Scientific, Cat# 88518) following the manufacturer’s recommendations. Membranes were blocked for one hour at room temperature with 5% dried skimmed milk in TBS (20 mM Tris–HCl pH 7.5, 150 mM NaCl) and then probed overnight at 4°C with specific antibodies diluted in TBS-T (TBS containing 0.1% Tween 20) containing 5% dried skimmed milk. Blots were probed with 1:300 rabbit anti-APC (#ab15490; Abcam), 1:1000 rabbit anti-NF-Kbeta p50, rabbit anti-Smad2/3 or mouse anti-Snail (#Sc-7178x, #Sc-133098, #Sc-271977 from Santa Cruz, respectively), or 1:3000 mouse/human anti-GADPH (ab9489; Abcam). The blots were then incubated with 1:3000 secondary antibodies either anti-rabbit (A0545) or anti-mouse (A9044) IgG-horseradish peroxidase-conjugated (Sigma-Aldrich) diluted in TBS-T for 1 h at room temperature.

The immune complexes were detected using ECL prime western blotting detection kit and a Uvitec Q9 alliance imaging system coupled to an Image Quant 10.2 software (Cytiva).

#### Immunostaining and fixed imaging studies

To determine the APC protein levels in LS174T colorectal cancer cell lines stably expressing APC-WT or APC-m4, cells were seeded on 18 mm^2^ round coverslips (#631-0153; VWR), fixed with warm 4% paraformaldehyde (#28908, Thermo Scientific) in Phosphate-Buffered Saline (PBS: 2.7 mM KCl, 1.8 mM KH_2_PO_4_, 10 mM Na_2_HPO_4_, 140 mM NaCl, pH 7.4) (#P4417 Sigma-Aldrich) for 15 minutes, washed once with PBS, permeabilized with 0.1% Triton X-100 (catalogue# T8787-100ML, Sigma-Aldrich) for 10 minutes, washed once with 1x PBST (1× PBS, 0.1% v/v Tween-20 : catalogue# P1379-100ML; Sigma-Aldrich), blocked with 3% BSA (#A3059-10G; Sigma-Aldrich) for 1 hour at room temperature and incubated overnight at 4°C with anti-APC C-terminus (1:250, Abcam, ab154906, RRID:AB_2861396). Coverslips were then washed with 1x PBST three times for 5 minutes each and incubated with secondary antibody Goat anti-rabbit Alexa-555 (1:1000; Cat# A-21428, RRID:AB_2535849, Thermo Fisher Scientific) along Alexa-488 Phalloidin (1:1000; A12379, Invitrogen-Thermo Fisher Scientific) to stain F-actin, for 1 hour at room temperature. Next, coverslips were washed with 1x PBST twice for 5 minutes each and mounted on glass slides (#N/A141, ACADEMY) using Aquamount mounting medium (#14-390-5, Thermo Fisher Scientific). Cells were imaged either in a Leica TCS SP8 SMD laser scanning microscope equipped with a EL6000 Fl light source, FOV scanner SP8, - 3.6 kHz tunable speed, 60x water objective (HC PL APO CS2 20x/0.75). Images were captured as stacks (11 planes, 0.5 μm steps) at 1-10% laser power using photomultiplier tube (PTM) detectors in sequential mode at 488 and 568 nm, and Pinhole: 1.00 AU.

To assess F-actin levels in collagen-embedded LS174T colorectal spheroids stably expressing APC-WT or APC-m4, spheroids were fixed with cold 4% paraformaldehyde (#28908, Thermo Scientific) in PBS (#P4417 Sigma-Aldrich) for 1 hour, washed twice with PBS, permeabilized with 0.5% Triton X-100 for 30 minutes, washed once with 1x PBST, blocked with 3% BSA for 1.5 hours at room temperature, and incubated for 4 hours with Alexa-488 Phalloidin at room temperature to stain F-actin. Next, spheroids were washed twice with PBS for 10 minutes. Images were acquired either in a Leica DMi8 or SP8 microscope with a 10x or a 20x objective (HC PL FLUOTAR 10X/0.45 or 20x/0.75) equipped with a Leica DFC9000GT camera. SP8 spheroid images were capture as stacks (30 planes, 0.5 μm steps) at 15-20 ms exposure. All images were registered in a LAS X Life Science Microscope Software (version 3.5.5.19976, RRID:SCR_013673).

#### Live-imaging studies

To investigate 3D spheroid formation, 500 cells - LS174T stably expressing APC-WT or APC-m4 – were seeded in Nunclon Sphera 96-well U-Bottom plates and imaged live (5 z-stacks with a 50 μm step) every 10 minutes for 16 hours. To investigate 3D spheroids invasion, collagen embedded spheroids were imaged live (5 z-stacks with a 50 μm step) for 1-4 hours and 24-28 hours post-embedding them in collagen.

#### Imaging data analysis

To analyze images, ImageJ/Fiji (v1.53k, RRID:SCR_003070/v2.3, RRID:SCR_002285), Microsoft Excel (v16, RRID:SCR_016137), GraphPad Prism (v9, RRID:SCR_002798) and Adobe Illustrator (v26.5, RRID:SCR_010279) were used.

##### Fluorescence intensity measurements

APC protein cellular densities were measured as in Baro et al. 2023. In brief, a custom macro was used to generate sum slices z-projections. Next, cell boundaries were manually drawn, and another custom macro was used to measure integrated density in Fiji.

To measure F-actin density in spheroids, F-actin was used as a marker to delineate spheroid boundaries of approximately 10 mm wide in ImageJ. Raw intensity and length of each trace were obtained in ImageJ and saved as .csv. The F-actin density at the spheroid boundaries was obtained by dividing the Raw intensity by the length of trace. Data was then analyzed and plotted in GraphPad Prism (version 9.3; GraphPad Software, La Jolla, CA).

##### Protrusion number, protrusion length, and spheroid area measurements

To measure number of protrusions per spheroid, .lif image files were opened in ImageJ and the “multi-point” tool was used to manually count the number of invasive protrusions per spheroid. To quantify protrusion length per spheroid, a line was manually drawn on each protrusion using the ‘‘segmented line’’ tool in Fiji and line length was measured using the ‘‘measure’’ tool. To measure spheroid areas, we first defined the ‘spheroid core area’ as the central group of interconnected cells and ‘invasive spheroid area’ as the attached area to the core but with cells shedding from the spheroid and migrating as leader cells away from the spheroid, i.e., excavating into the collagen matrix. Lines were manually drawn around each spheroid area using the “segmented line” tool. Data was compiled in Microsoft Excel (v16, RRID:SCR_016137) and then analyzed and plotted in GraphPad Prism (version 9.3; GraphPad Software, La Jolla, CA) displayed as violin plots (for number of protrusions and spheroid core area) or as Superplots^66^^,^^67^ (for protrusion length).

##### Tracking of migrating cells emanating from invasive protrusions

To track individual cells emanating from spheroids pre-embedded in collagen matrix for 24 hours, we used the “manual tracking” tool in Fiji. The coordinates were saved as a .txt file and exported into Chemotaxis and Migration Tool version 2.0 software (Ibidi, Germany RRID: SCR_022708) to obtain migratory related parameters such as the directionality, velocity [μm/min], Euclidean Distance [μm] – defined as the shortest distance between start and end cell location, and Accumulated Distance [μm] of each cell tracked. The obtained data was then analyzed and plotted in GraphPad Prism. Finally, single cell tracks were plotted using celltrackR^68^ and ggplot2 in RStudio using a custom R script which is available in Zenodo (https://doi.org/10.5281/zenodo.8387899).

For invasive spheroid area measurement, total spheroid and core spheroid boundaries were manually drawn and the area was measured in Fiji. The normalized areas were calculated as follows:

Normalizedinvasivearea=(Totalspheroidarea−corespheroidarea)/corespheroidarea

Normalizedcorespheroidarea=(corespheroidarea−corespheroidareaatt0)/corespheroidareaatt0

Data was then analyzed and plotted in GraphPad Prism.

### Quantification and statistical analysis

All experiments were repeated multiple times, as indicated in figure legends. Data were pooled and, if required, analyzed further in Microsoft Excel (v16), and plotted in GraphPad Prism (v9.3; GraphPad Software, La Jolla, CA). Single-cell tracks were plotted in RStudio (v4.2) using ggplot2. Figure legends specify the n, errors, and the statistical test used. Data distributions were tested for normality using the D'Agostino-Pearson omnibus normality test, and statistical differences among conditions were calculated using ordinary one-way ANOVA with Tukey multiple comparisons tests, Welch’s t-test or Mann-Whitney U test (non-parametric) in GraphPad Prism (v9.0; GraphPad Software, La Jolla, CA). Superplots displayed the mean of the different replicates and superimposed the distribution of ‘n’ (as color-code dots) as violin plot.^66^^,^^67^ Mean from the different replicates, standard deviation and paired two-tailed t-test were used to find the statistical differences between replicates. This way of representing data linked paired measurements together and conveyed the repeatability of the work, eliminating the need to normalize data to directly compare different experimental replicas. Differences were considered significant if p-value was <0.05 (∗), <0.01(∗∗), <0.001(∗∗∗), or < 0.0001 (∗∗∗∗), as indicated in each figure legend. In some cases, to save space, p-values ranging from p<0.001 to 0.05 were represented either as ‘a’ (when a significant difference relative to APC-WT control (+DMSO) was found); or as ‘b’ (when a significant difference between combinations in APC-m4 but relative to APC-m4 control (+DMSO) was found).
