# Supplementary material for: Tumor invasiveness is regulated by the concerted function of APC, formins, and Arp2/3 complex
Source: iScience. 2024 Apr 8;27(5):109687. doi: 10.1016/j.isci.2024.109687 (PMC11053316; doi:10.1016/j.isci.2024.109687)
Supplement: Document S1. Figures S1–S3 [file mmc1.pdf]

iScience, Volume *27*

Supplemental information

Tumor invasiveness is regulated by the concerted function of APC, formins, and Arp2/3 complex

Lautaro Baro, Rabeah A. Almhassneh, Asifa Islam, and M. Angeles Juanes

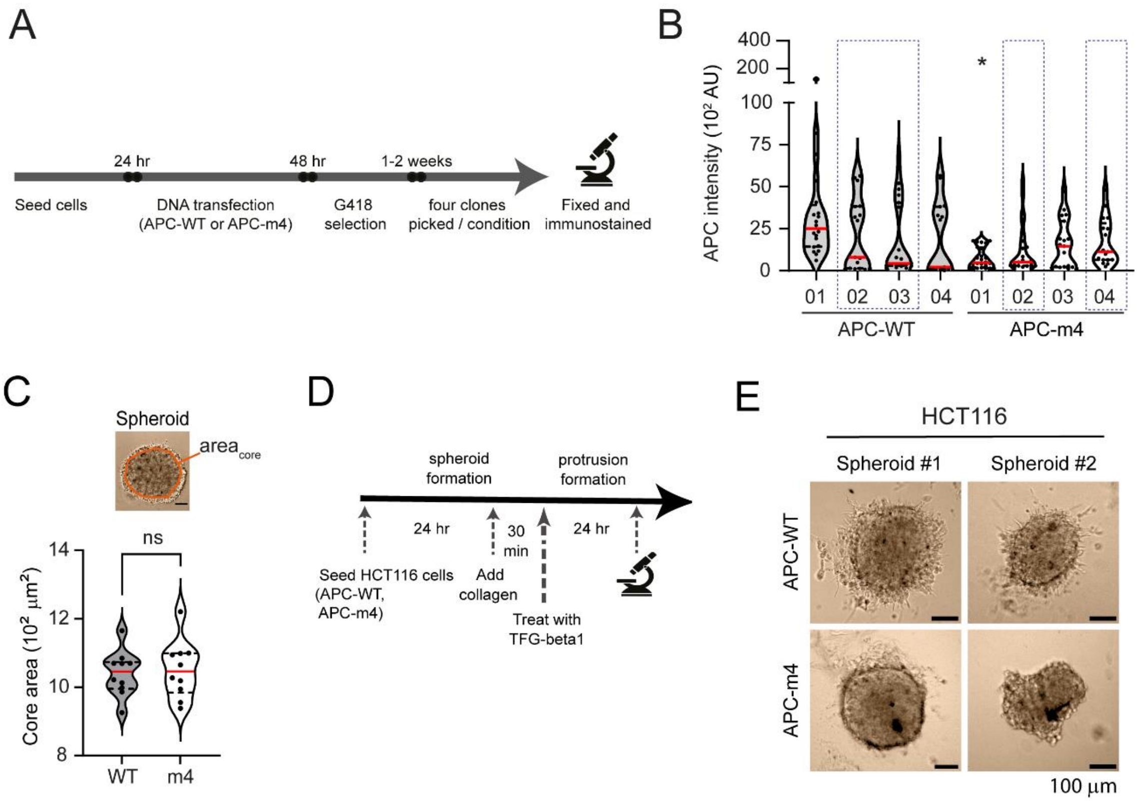

Figure S1. Related to Figure 1. Generation of LS174T stable colorectal cancer cell lines, and effects in LS174T and HCT116 spheroids. Data from A-C are from LS174T cells stably expressing APC-WT or APC-m4, data from D-E are from HCT116 cells stably expressing APC-WT or APC-m4. **(A)** Experiment regimen. **(B)** Violin plot showing APC fluorescence signal in the corresponding clones (C01-04). N = 3 replicates, n cells: APC-WT:C01 = 23, APC-WT:C02 = 19, APC-WT:C03 = 21, APC-WT:C04 = 17, APC-m4:C01 = 24, APC-m4:C02 = 23, APC-m4:C03 = 19 and APC-m4:C04 = 17. Data is from two independent repeats. The solid line is the median and the dotted lines are quartiles. One-way ANOVA with Tukey correction was performed to find the statistical differences using APC-WT:C02 as a control. **(C)** Outline of the core area as measured in spheroid images to generate a violin plot for core area of spheroids after 8 hours from embedding in collagen. N = 3 replicates, n = 10 spheroids per condition. Data are from three independent repeats. The red solid line is the median and the dotted lines are quartiles. Student t-test with Welch correction was performed to find the statistical differences. 'ns' is not significant. **(D)** Experiment regimen. **(E)** Representative images showing spheroids embedded in collagen and treated with TFG-beta 1, as shown in D. Scale bar = 100 µm.

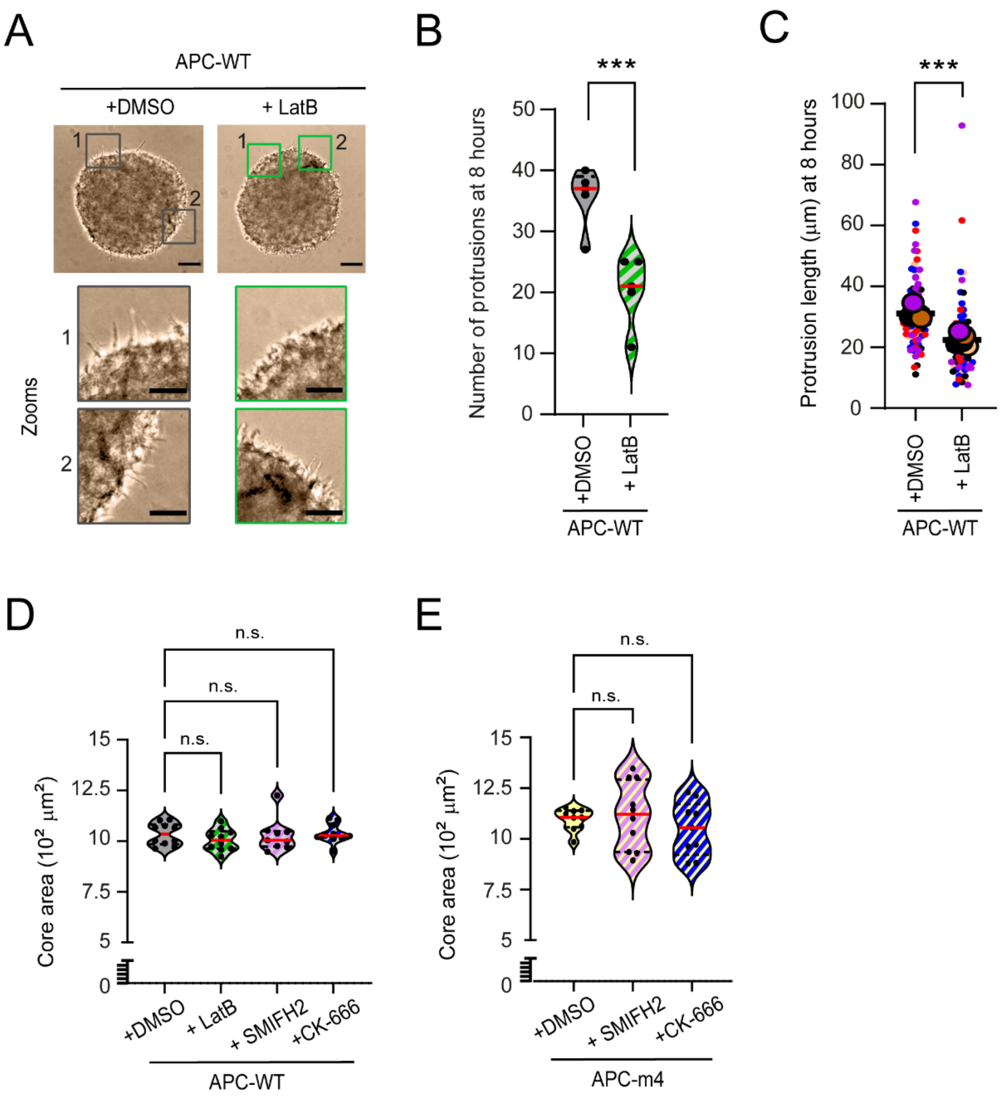

Figure S2. Related to Figure 2. Effects of perturbation of certain actin cytoskeleton networks. All data are from LS174T cells stably expressing APC-WT or APC-m4, untreated or treated as indicated. **(A)** Representative images showing spheroids embedded in collagen and treated with Latrunculin B, an actin inhibitor. Insets correspond to selected sections of the spheroid periphery to view protrusions after 8 hours of inhibitor treatment. Scale bar = 100 µm, inset = 50 µm. **(B)** Violin plot showing the number of protrusions per spheroid from images obtained as in Figure 2A-B. N = 3 replicates. The solid red line is the median. Statistical significance was derived from the Mann-Whitney U test. '***' is $p<0.001$. **(C)** Violin plot showing the average length of invasive protrusions. N = 5 independent replicates for each condition; n is individual protrusions used to quantify length: APC-WT+ DMSO = 83, APC-WT + Latrunculin-B = 77. Data are displayed as 'Superplots' showing the mean of the different replicates (circles) and the distribution of 'n length of protrusion analyzed' (color-coded dots) was superimposed as violin plot. Black solid line is the mean and paired two-tailed t test was used to find the statistical differences using N = 5 replicates. Statistical significance was derived from the Mann-Whitney U test. '***' is $p<0.001$. **(D-E)**

Violin plots showing core area of spheroids after 8 hours from embedded spheroids from images as in Figure 2A-B. N = 3 replicates; n = 5 spheroids per condition. The red solid line is the median. Student t-test with Welch correction was performed to find the statistical differences. 'ns' is not significant.

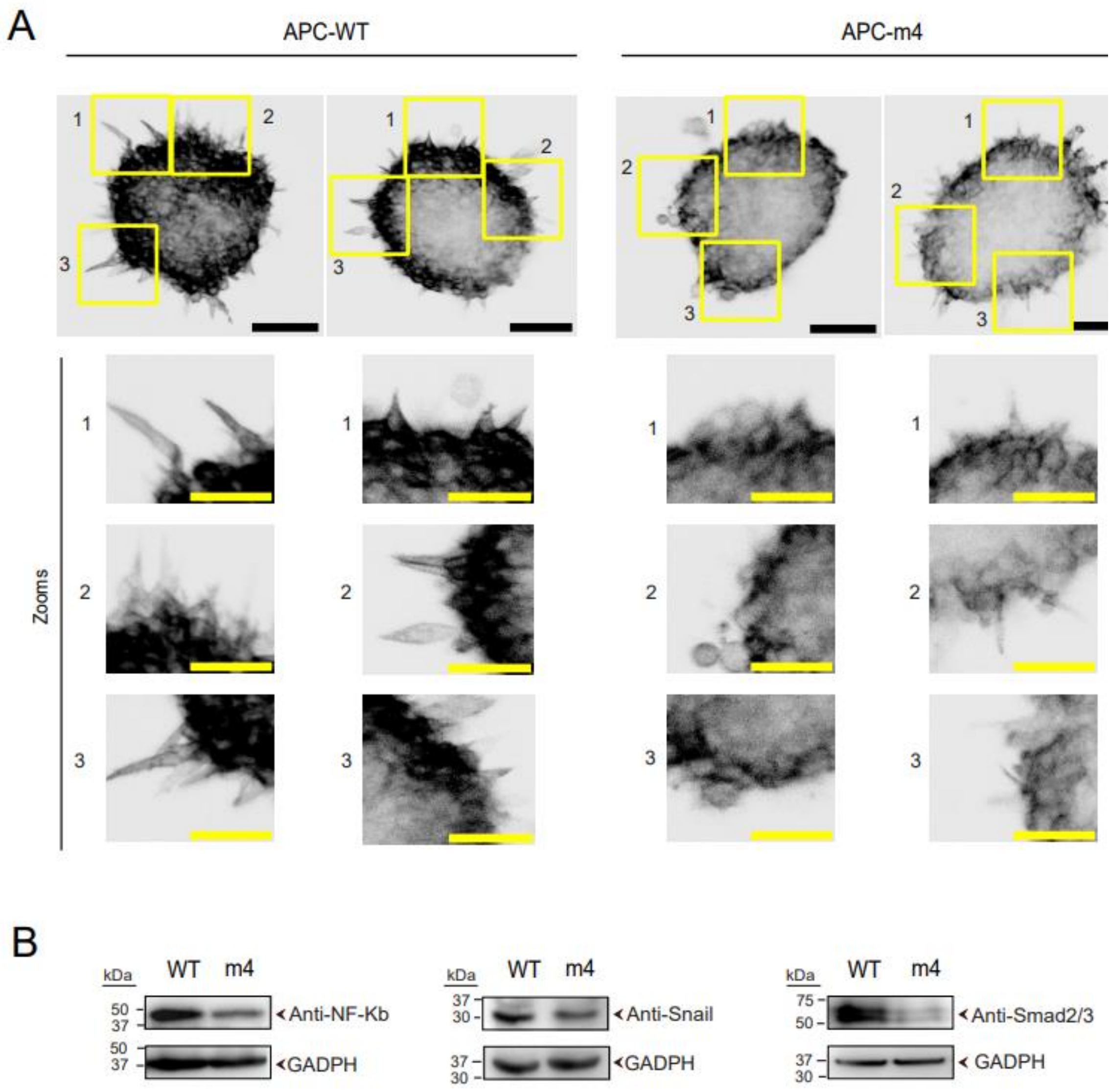

Figure S3. Related to Figure 4. Effects of the APC-m4 on actin staining in spheroids and in key proteins from signaling pathways. All data are from LS174T cells stably expressing APC-WT or APC-m4. **(A)** Representative images showing spheroids (two per condition) embedded in collagen and stained with SiR-actin dye to visualize F-actin using a confocal microscope. Insets correspond to sections of the spheroid periphery to view protrusions after 21-24 hours from staining. Scale bar = 100 µm, inset = 50 µm. **(B)** Representative western blots showing protein levels of NF-kb, Snail and Smad2/3 obtained from whole-cell extracts. GADPH was used as a loading control.
